# Radiolabeled Vitamins and Nanosystems as Potential Agents in Oncology Theranostics: Developed Approaches and Future Perspectives

**DOI:** 10.3390/jpm16010036

**Published:** 2026-01-05

**Authors:** Ghazal Basirinia, Albert Comelli, Pierpaolo Alongi, Muhammad Ali, Giuseppe Salvaggio, Costanza Longo, Domenico Di Raimondo, Antonino Tuttolomondo, Viviana Benfante

**Affiliations:** 1 Department of Health Promotion, Mother and Child Care, Internal Medicine and Medical Specialties, Molecular and Clinical Medicine, University of Palermo, 90127 Palermo, Italy; gbasirinia@fondazionerimed.com (G.B.); domenico.diraimondo@unipa.it (D.D.R.); bruno.tuttolomondo@unipa.it (A.T.); 2 Ri.MED Foundation, Via Bandiera 11, 90133 Palermo, Italy; amuhammad@fondazionerimed.com; 3 NBFC—National Biodiversity Future Center, 90133 Palermo, Italy; 4 Advanced Diagnostic Imaging-INNOVA Project, Department of Radiological Sciences, A.R.N.A.S. Civico Di Cristina e Benfratelli Hospitals, P.zza N. Leotta 4, 90127 Palermo, Italy; pierpaolo.alongi@unipa.it; 5 Department of Biomedicine, Neuroscience and Advanced Diagnostics (BiND), University of Palermo, 90127 Palermo, Italy; giuseppe.salvaggio@policlinico.pa.it; 6 Nuclear Medicine Unit, A.R.N.A.S. Civico Di Cristina e Benfratelli Hospitals, P.zza N. Leotta 4, 90127 Palermo, Italy; costanza.longo@arnascivico.it

**Keywords:** targeted radionuclide therapy (TRT), vitamin-related radiopharmaceuticals, vitamin, B9, folate, B12, theranostics, nanoparticles

## Abstract

Theranostic approaches employing radioactive materials have emerged as innovative strategies that integrate molecular imaging with targeted therapy using nanosystems, thereby advancing the paradigm of precision medicine in oncology. Each year, substantial research efforts are dedicated to developing molecular probes capable of detecting early-stage tumors, with improved efficacy and reduced toxicity to the surrounding healthy tissues. Radiopharmaceuticals based on vitamins and nanoparticles are among the most promising developments in this field, as they possess a high level of specificity and low toxicity. Vitamin B9 and vitamin B12 represent notable examples, as their targeting properties exploit the overexpression of corresponding receptors in tumor cells. In this context, future directions may include the radiolabeling of nanoparticles functionalized with these vitamins using isotopes such as [^68^Ga] and [^177^Lu], thereby enabling both diagnostic imaging and therapeutic applications. Despite the encouraging preclinical evidence, many in vitro and in vivo studies employing these strategies do not sufficiently address their translational applicability to radiotheranostics. This review highlights the most promising advances in the diagnostic and therapeutic potential of vitamin and nanoparticle-based systems. It aims to critically evaluate current findings and propose hypotheses for further study in the emerging field of radiopharmaceutical theranostics.

## 1. Introduction

Precision oncology theranostics combines diagnostic imaging with targeted therapies. This approach relies on nuclear medicine to identify and target tumor-specific biomarkers using positron emission tomography (PET) and single-photon emission computed tomography (SPECT) [1].

PET and SPECT are functional imaging techniques that utilize radioactive tracers. PET employes positron-emitting isotopes such as (^18^F, ^15^O, and ^11^C), whereas SPECT uses gamma emitters including (^123^I and ^99m^Tc). Superior resolution and sensitivity offer with PET, but it is more expensive, while SPECT is more affordable and widely available [2,3].

Targeted radionuclide therapy (TRT) is a nuclear medicine approach that delivers ionizing radiation directly to cancer cells by exploiting the specific accumulation of radiolabeled compounds at the tumor site [4].

Radiopharmaceuticals are designed using selected radionuclides and [4,5] biomolecules to achieve effective tumor targeting, optimizing factors such as half-life and energy to enhance tumor destruction while minimizing resistance [6]. Each radiopharmaceutical consists of two key components: a carrier molecule or ligand, which facilitates targeted delivery, and a radionuclide, whose nuclear properties determine its diagnostic or therapeutic function [7]. Carriers play a pivotal role in the precise delivery of radionuclides to specific biological targets. Chelators are essential for stabilizing metallic radionuclides such as ^99m^Tc and ^66/68^Ga, while linkers may be incorporated to improve pharmacokinetics and binding affinity [8,9]. The proper selection of these elements is critical for maximizing therapeutic efficacy while minimizing biological damage [10].

**Figure 1:**
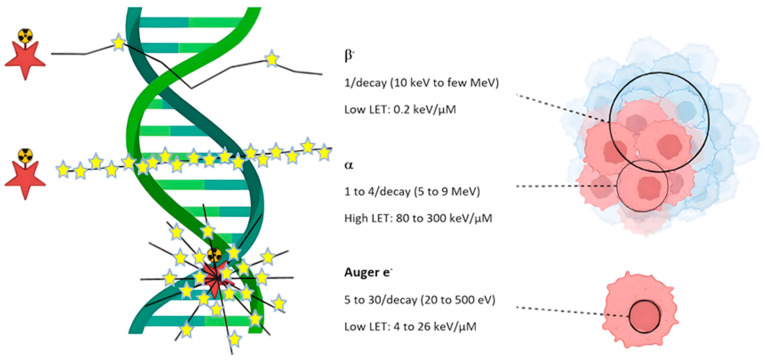
Linear Energy Transfer (LET) for various particle types, including beta-minus (β^−^) particles, alpha (α) particles, and Auger electrons. Red stars are the radiation source and yellow stars show disintegrations. This figure depicts the effects of these types of radiation on DNA and biological structures highlighting differences in their energy and ionization patterns. β^−^ radiation, has low linear energy transfer (LET ~0.2 keV/μm), causes widespread but relatively mild damage. α radiation, with high LET (80–300 keV/μm), creates dense, localized ionization due to significant damage to DNA and cells. Despite the low energy of Auger electrons with (20–500 eV), produce highly concentrated ionization with severe damage in areas near their emission, particularly, around DNA. Thus, when the LET increases, the intensity of damage becomes greater, while the affected area decreases. Reprinted from Ref. [11], Copyright 2023, Pharmaceutics. This article is an open access article distributed under the terms and conditions of the Creative Commons Attribution (CC BY) license.

Radionuclides, as inherently unstable atoms, undergo radioactive decay during which they emit alpha particles, beta particles, Auger electrons, or gamma rays. Because of their energy and ionizing properties, these emissions can induce irreversible DNA damage in cancer cells, ultimately triggering apoptosis (Figure 1) [11]. Beta and alpha emitters are the primary components of radionuclide-based treatments, while Auger emitters are used less commonly. The development of carrier molecules has expanded the clinical use of beta emitters, such as yttrium-90 [^90^Y] and lutetium-177 [^177^Lu]. Although alpha emitters have been utilized for several decades, their short tissue penetration and highly localized radiation delivery have recently increased their appeal for treating small tumors [12]. Therefore, careful selection of the appropriate radionuclide is essential to maximize therapeutic benefits while ensuring radiation safety [13].

Molecular imaging has emerged as a transformative diagnostic modality, offering superior sensitivity and spatial resolution compared with conventional imaging techniques. The integration of molecular imaging with highly specific and biologically active tracers enables the visualization and quantification of pathological processes at the cellular and molecular levels. This allows for detection of small or otherwise indiscernible lesions. This capability holds significant implications for oncology, where early identification of malignant changes, accurate tumor staging, and real-time assessment of therapeutic response are crucial for optimizing patient outcomes [14]. The key advantages of using vitamins in radiopharmaceutical design are their low cost, non-toxicity, accessibility, and proven anti-cancer properties [15]. In cancer diagnosis, targeted radionuclide imaging has shown promising results. Radiolabeled vitamins for cancer SPECT and PET represent a flexible approach in both preclinical and clinical research [16,17]. Notably, they have demonstrated effective results in the diagnosis of various cancers, including breast, ovarian, lung, and colon cancers. A major challenge in this approach is the synthesis of suitable vitamin-metal conjugates to minimize unintended radiotracer accumulation in non-target tissues and reduce adverse effects. Additional challenges involve achieving efficient transport, specific uptake, and retention of radiolabeled vitamins in tumor tissues. Thus, Vitamin based radiopharmaceuticals are expected to play a key role in cancer diagnosis and therapy, becoming indispensable tools in nuclear medicine [18].

Monoclonal antibodies, small peptides, and nanoparticles smaller than 100 nanometers can be radiolabeled with specialized radionuclide for theranostic applications. Prostate-specific membrane antigen (PSMA) is one of the most widely used biomarkers in radiotheranostics; However, since it is also overexpressed in other tumors, radioprotective strategies for healthy organs are essential [19,20]. Given that vitamins are physiological components of the human body, it is interesting to explore their potential involvement in the development and testing of new radiolabeled compounds for both diagnosis and therapy, from a radiotheranostic perspective. The idea is to collect state-of-the-art materials to convey information regarding the use of therapeutic radionuclides, such as Lutetium-177, and diagnostics such as Gallium-68, tumor biomarkers, along with tumor biomarkers and vitamins employed in nanoscale formulations for precision oncology.

Current cancer treatments include chemotherapy, radiation therapy, immunotherapy, surgery, and various combinations of these approaches. Despite their benefits, conventional chemotherapeutics are non-selective and can damage healthy tissue, leading to unwanted side effects. This can contribute to multidrug resistance, further limiting in therapeutic options for most aggressive forms of cancer, such as metastatic cancer [21].

Nanotechnology-based compounds exploit nanocarriers designed to encapsulate therapeutic agents, such as chemotherapeutics or radionuclides. Their structure allows for extremely precise targeting of tumor cells and, thanks to their biocompatibility, protection of the surrounding tissues. Passive targeting exploits the permeability of blood vessels in the tumor microenvironment to promote nanocarrier accumulation on the lesion. Active targeting involves decorating nanocarrier surfaces with specific biological ligands that attach to tumor cells, enhancing specific treatment [22].

Nanotechnology scaffolds hold promise for improving therapeutic efficacy, reducing side effects in healthy tissues, and thereby enhancing patient safety. However, despite the experimental evidence, only a few nanocarriers tested in animal models advance to clinical trials. This could be due to challenges in conversion of data from the in vitro and preclinical phase into clinical dosimetry and cytotoxicity assessments [23].

In healthy tissues, folate receptors are minimally expressed and primarily localized to cells involved in embryonic development and folate homeostasis. In contrast, under certain pathological conditions, folate receptors are overexpressed in specific tissues, presenting opportunities for targeted therapeutic intervention. Folate receptors can be targeted by folic acid or other high-affinity folate receptor (FR) ligands and anti-FR monoclonal antibodies [24].

In addition to being the cost-effective strategy, folic acid is conjugated to the surface of drugs or nanoparticles, usually via a linker such as polyethylene glycol [25]. The use of nanoformulations containing anti-FR monoclonal antibodies has also been extensively studied in oncology, allowing FR to be targeted more specifically to sites of inflammation or macrophages within the tumor microenvironment [26]. A promising application of folate receptor targeting is in ovarian cancer treatment and imaging, as the majority of epithelial ovarian carcinomas overexpress folate receptors, accounting for nearly 95% of ovarian cancer deaths [27]. Increasing vitamin B12 values can be indicative of the presence of malignancies, in particular hematological diseases, reflecting the presence of proliferating cells and immune responses associated with cancer [28].

Folate and vitamin B12 are partially associated with the same pathway, specifically the methyl trap hypothesis and thus low levels of methionine synthase. This pathway could be targeted to increase the binding and activation of therapeutic agents in tumors characterized by folate and vitamin B12 [29]. In the near future, it would be interesting to radiolabeling nanoparticles based on these vitamins with [^68^Ga] and [^177^Lu] for diagnosing and treating patients. However, many preclinical in vitro and in vivo studies employ these approaches without addressing their applicability in radiotheranostics.

This review highlights the most promising results in diagnostics and therapy of these systems, critically trying to outline hypotheses that can be investigated in the near future in the field of radiopharmaceutical theranostics.

## 2. Methods

This review was conducted in order to provide a critical assessment of the current state of preclinical research on vitamin-based radiopharmaceuticals in oncology. To ensure a comprehensive, transparent, and unbiased synthesis of the evidence, a systematic approach was employed, guided by the PRISMA-ScR (Preferred Reporting Items for Systematic reviews and Meta-Analyses extension for Scoping Reviews) [30] framework where applicable. The protocol for this scoping review was not registered in a publicly accessible database.

### 2.1. Search Strategy and Information Sources

A systematic literature search was performed across three major electronic databases: PubMed, Scopus, and Google Scholar. The search strategy was designed to encompass the core concepts of the review: cancer types, radionuclide therapy, theranostics, vitamins, and nanotechnology. The following search string was adapted for each database: (“Gastric Cancer” OR “Breast Cancer” OR “Colorectal Cancer” OR “Brain Cancer” OR “Prostate Cancer” OR “Ovarian Cancer”) AND (“Targeted Radionuclide Therapy” OR TRT OR Theranostic) AND (“Vitamin B9” OR Folate OR “Folic Acid” OR “Vitamin B12” OR Cobalamin) AND (Radiopharmaceutical OR Radionuclide OR Nanoparticle). The search was limited to studies published in English between 1 January 2000 and 31 October 2025.

### 2.2. Eligibility and Exclusion Criteria

Studies were included if they met the following criteria: (1) original research articles or reviews investigating vitamin-based radiopharmaceuticals; (2) included in vitro and/or in vivo preclinical models; (3) focused on diagnostic or therapeutic applications in oncology. Exclusion criteria were: (1) non-English publications; (2) conference abstracts, editorials, or patents; (3) studies not involving a radiolabeled vitamin or vitamin-conjugated nanosystem; and (4) studies focused solely on clinical case reports without novel preclinical data.

### 2.3. Study Selection and Data Extraction

**Figure 2:**
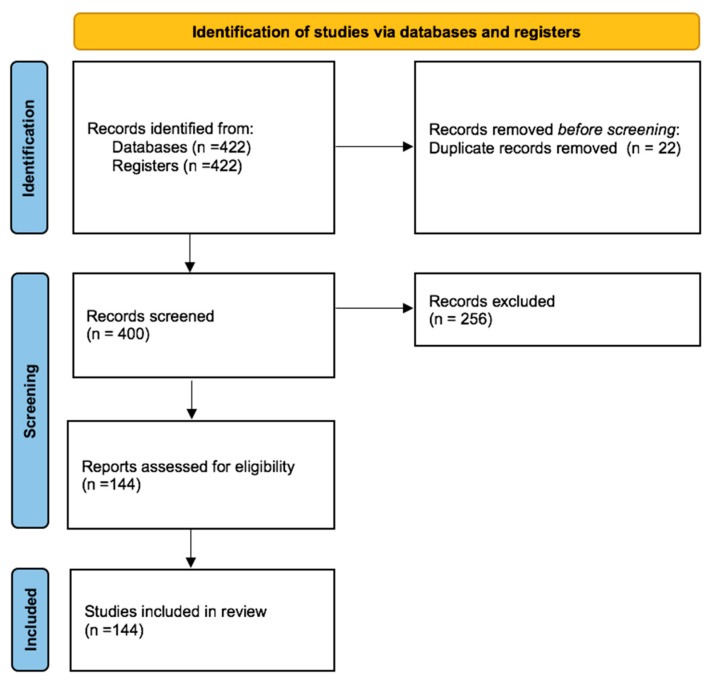
PRISMA Flow Diagram of the Study Selection Process.

The study selection process is summarized in a PRISMA-style flow diagram (Figure 2). After removing duplicates, titles and abstracts were screened for relevance. Full-text articles that met the initial criteria were then retrieved and evaluated against the predefined inclusion and exclusion criteria. Any differences in study selection were resolved through discussion, with additional input sought when necessary. Data from the eligible studies were extracted into a standardized spreadsheet, capturing information such as the first author, publication year, cancer model, radionuclide, targeted agent (vitamin conjugate), key findings (including uptake values, imaging performance, and therapeutic outcomes), and the vitamin utilized. For more information, please refer to the Supplementary Materials.

- Identification: Records identified from databases (n = 422).
- Screening: Records screened after duplicates removed (n = 400).
- Eligibility: Full-text articles assessed for eligibility (n = 144).
- Included: Studies included in the qualitative synthesis (n = 144)

## 3. Gastric Cancer-Targeted Vitamin-Based Radionuclide Agents

Gastric cancer (GC) represents the fourth leading cause of cancer-related mortality worldwide and continues to be among the most prevalent malignancies, with an estimated 400,000 new cases diagnosed annually [31,32]. GC constitutes a major global health challenge, with its burden being especially pronounced in East Asian populations. The disease is characterized by both a high incidence rate and an overall poor prognosis, outcomes that are largely attributable to the fact that the majority of cases are diagnosed at advanced stages when therapeutic options are limited and curative treatment is less feasible. Consequently, the late detection of GC not only worsens survival outcomes but also amplifies its impact as a critical public health concern in the region [33,34]. While WHO classifications exist, an effective biological classification for clinical use is still lacking [35]. Primary treatments, such as surgery and chemotherapy, as well as other therapies, including radiotherapy, immunotherapy, and targeted therapy, are investigated. Despite these options, the survival rate is low, less than 42% for metastatic cancers [36]. Therefore, the urgent need for novel therapeutic strategies is crucial to reducing GC-related mortality. GC is characterized by a heterogeneous cell population that drives malignancy through dysregulated growth, differentiation, angiogenesis, migration, and metastasis in a highly complex carcinogenic process [37,38]. GC pathogenesis is strongly influenced by environmental factors, with Helicobacter pylori infection, diet, and smoking being significant risk factors [39]. Analysis of genomic data of GC tissues indicating four molecular subtypes: EBV, MSI, GS, and CIN which each one has shown different features like extreme DNA hypermethylation for EBV, high mutation rates for MSI, low fewer genomic alterations for GS and marked aneuploidy also receptor tyrosine kinase amplifications in CIN subtype, which this may guide the personalized treatment potential regarding these features impacted on the therapeutic strategies [40]. Additionally, Epstein–Barr virus (EBV) infection has been identified as a potential etiological agent in 5–10% of GC cases [41]. The use of biomarkers in GC diagnosis and treatment, particularly those targeted integrin receptors, HER2, Claudin 18, and glutathione-responsive systems, has driven significant advancements in targeted therapies and nanotechnology. The future of GC management is expected to advance toward personalized medicine, wherein biomarker-based strategies with their potential to enhance the optimization of targeted radionuclide therapies, offer hope for improved treatment outcomes [42].

Takashima et al. investigated a ^211^At-labeled anti-TF monoclonal antibody (clone 1084) in both in vitro (MKN-1, MKN-45, NUGC-3 cell lines) and in vivo (xenograft mouse) gastric cancer models. Tissue Factor specifically targets Clone 1084, with the therapeutic efficacy of the ^211^At–anti-TF conjugate through precise accumulation in TF-positive tumors and localized delivery of high-LET α-radiation. They showed that sodium ascorbate, a free radical scavenger, prevented ^211^At-induced antibody denaturation, preserving its antitumor activity. Though vitamin-derived, sodium ascorbate served as a stabilizer, not a targeting agent, enhancing therapeutic safety and efficacy [43].

Moreover, Muller et al. presented [^177^Lu]-labeled folate conjugates incorporating an albumin-binding component, known as cm09, to increase blood circulation and optimize the rate of tumor-to-kidney uptake. High radiolabeling efficiency (>98%) and strong plasma protein binding (91%) were demonstrated by [^177^Lu]Lu-cm09. Additionally, it showed specific tumor uptake (17.56% ID/g at four h) with reduced kidney accumulation. Biologically, the albumin-binding component extends systemic circulation through reversible interaction with serum albumin, which reduced renal filtration and folate receptor, effectively lowering kidney accumulation without compromising tumor-specific uptake. SPECT imaging displayed significant tumor-to-background ratios in mice with KB tumors. Specifically, these conjugates exhibited a tumor-to-kidney ratio that was 5 to 6 times higher than those without the albumin binder, which resulted in extended survival [44].

Si and colleagues evaluated three different bioactive molecules labeled with [^68^Ga], namely [^68^Ga]Ga-DOTA-RGD, [^68^Ga]Ga-DOTA-FA, and [^68^Ga]Ga-DOTA-BSA, which were prepared using new approaches under mild conditions. The preclinical studies were conducted using human gastric cancer (MGC-803) and ovarian carcinoma (SKOV-3) cell lines, as well as tumor-bearing mice, as animal models. Synthesizing these radiotracers using new strategies has shown great biological and chemical functions, also demonstrating high affinity and stability. Thus, all labeling processes, such as chelation, linking, and purification, were performed in under 40 min to maintain molecular bioactivity; due to this efficient and significant approach, they approved the promising potential of [^68^Ga]-DOTA-Mal in providing novel PET tracers and advancing PET imaging technologies [45].

The development of [^68^Ga]Ga-DOTA-folate, [^68^Ga]Ga-NODAGA-folate, and [^68^Ga] Ga-NOTA-folate displayed significant advancement in creating PET radiotracers targeting FR-positive tumors. Each compound was designed to enhance tumor specificity, stability, and imaging performance using specific chelators (DOTA, NODAGA, and NOTA) to complex gallium-68. Preclinical studies highlighted their strong affinity for FRs and effective tumor uptake, with PET imaging showing high imaging contrast and biodistribution profiles. Importantly, competitive blocking with excess folic acid or EC20 was utilized in multiple models, which confirmed target specificity and temporary receptor saturation in normal tissues, particularly renal FR-α, can reduce retention of the kidney without significantly tumor accumulation effect. These findings emphasize their potential as diagnostic agents for FR-positive cancers and provide valuable tools in non-invasive imaging and precision medicine, also highlighting the clinical relevance of co-infusion approaches and informing organ-at-risk dosimetry considerations for future therapeutic folate conjugates [46,47,48].

Additionally, a new [^68^Ga]Ga-NOTA-folic acid conjugate developed by Jain et al. is used for PET imaging of tumors that overexpress folate receptors and for evaluating its potential in human nasopharyngeal carcinoma cell lines with overexpressed folate receptors, as well as for preliminary biodistribution studies in normal Swiss mice. The synthesis was successful, and radiolabeling was performed with an efficiency of over 95% and a binding affinity to cells. It showed specific uptake by KB cells, which was blocked by cold folic acid, and holds potential applicability in other FR-positive cancers, such as gastric cancer. The biodistribution study of the [^68^Ga]Ga-NOTA-folic acid conjugate in normal mice showed a strong correlation between the tracer and kidneys. Additional studies in tumor models are required to validate its imaging potential with future optimization focused on reducing renal dosimetry risk to enhance its clinical applicability [49].

Lu et al. reported an in vitro and in vivo evaluation of newly synthesized folate conjugates, HYNIC-NHHN-FA, radiolabeled with [^99m^Tc], which used different coligand systems. They analyzed the biological profile of these radiotracers in KB tumor cells, which are folate receptor (FR)-positive. The biodistribution study in mice models demonstrated that the radiocomplex [^99m^Tc]Tc-(HYNIC-NHHN-FA) (tricine/TPPMS) was primarily absorbed in the stomach and intestines, indicating rapid elimination of radioactivity through the gastrointestinal system due to low tumor uptake, suggesting that optimizing coligand chemistry significantly influences both pharmacokinetics and tumor-to-background contrast. As a result, [^99m^Tc]Tc-(HYNIC-NHHN-FA) (tricine/TPPTS) indicates a promising candidate for FR imaging [50]. Another experiment by Xie et al. focused on FR-positive KB tumors as the target and developed [^99m^Tc]Tc-labeled folate-based SPECT radiotracer. To optimize binding affinity, they utilized HYNIC as a chelator and polyethylene glycol (PEG) as a linker. The radiotracer [^99m^Tc]Tc-(HYNIC-PEG2-FA) (EDDA) exhibited moderate uptake in the gastrointestinal tract, suggesting its involvement in excretion. Additionally, a significant accumulation of [^99m^Tc]Tc-folate was observed in the kidney. The administration of excess folic acid significantly reduced radiotracer uptake in both tumors and kidneys, confirming receptor-specific targeting and showing the biological basis of competitive receptor saturation as a blocking strategy to reduce renal exposure [51].

Kim et al. designed a novel [^99m^Tc]Tc-ECG-EDA (Glu-Cys-Gly-ethylenediamine)-folate as an FR-positive KB tumor imaging agent, utilizing a mouse tumor model for in vivo studies. ECG-EDA-folate was radiolabeled with [^99m^Tc], exhibiting high efficiency and stability (>96%), which demonstrated specific targeting of FR-positive tumors, as confirmed by micro-SPECT/CT imaging and reduced uptake with excess folate. These findings provide valuable insights for developing advanced folate-peptide conjugates for tumor imaging applications [52].

Boss et al. synthesized and compared three pairs of α- and γ-conjugated fluorinated folate derivatives, which were radiolabeled with fluorine-18 [^18^F]. The process of radiolabeling yielded high purities of radiochemicals (>95%) and a range of significant activities from 25 to 196 GBq/μmol. The in vitro assay demonstrated a strong FR-α binding affinity in all derivatives, with IC50 values ranging from 1.4 to 2.2 nM. In vivo studies in FR-positive tumor-bearing mice, regressing to PET imaging and biodistribution assays, demonstrated that sites of conjugation impacted biodistribution due to non-specific liver uptake of α-regioisomers compared to γ-regioisomers, which exhibited reduced kidney uptake of up to 50%. Thus, the results underscore the critical role of radiolabeling site selection in designing [^18^F]-based radiofolates as a novel way for advanced folate-based radiotracer designs [53].

Betzel et al. investigated a new radiotracer, 3-aza-2-[^18^F]F-fluorofolic acid, for imaging FR-positive tumors. In vitro testing demonstrated strong FR-α binding affinity (IC_50_ ≈ 0.8 nM). In vivo PET imaging of FR-positive KB tumor-bearing mice revealed substantial tumor uptake (≈12.6% ID/g at 90 min post-injection). High accumulation was also observed in the kidneys (≈57% ID/g) and salivary glands (≈14% ID/g), both of which are organs known to express physiological levels of FR. Additionally, minor non-specific uptake occurred in the liver. Thus, the tracer demonstrated favorable in vitro and in vivo properties and enabled high contrast and minimal off-target accumulation, which showed notable advantages with its efficient synthesis process and clinical potential [54]. Additionally, a new radiolabeled folic acid derivative (2′-[^18^F]F-fluorofolic acid) has been developed in a preclinical study by Ross et al. that showed high affinity and specificity for folate receptors. This radioligand outperforms previous [^18^F]-labeled radiofolates in terms of receptor selectivity and, to date, is considered to be the most promising [^18^F] radioligand for imaging folate receptor-positive tumors, due to integrating synthetic efficiency, minimal off-target accumulation, and favorable organ dosimetry, which are essential attributes for the future clinical translation of FR-targeted cancer imaging [55]. The other radiotracer, [^18^F]F-fluorodeoxyglucose-folate, was synthesized by Fisher and colleagues, who used a glucose-based radiolabeled prosthetic group to enhance hydrophilicity. High affinity and specificity for folate receptors due to in vitro and in vivo studies, demonstrated the potential of this radiotracer for PET imaging of FR-positive cancers and inflammation [56] and its efficient use for clinical translation [57].

## 4. Breast Cancer-Targeted Vitamin-Based Radionuclide Agents

Breast cancer (BC) is a malignant disorder that remains a major global health concern for women due to its high incidence and mortality. Its development is influenced by several risk factors, including genetic and hereditary predispositions. Advances in treatment strategies have increasingly focused on molecular characteristics, such as HER2 activation, estrogen receptor (ER) and progesterone receptor (PR) expression, gene mutations, and tumor microenvironment markers [58]. Early diagnosis of breast cancer significantly improves prognosis and patient survival. However, conventional imaging techniques often lack the sensitivity to detect tumors before morphological changes become apparent. In contrast, radionuclide-based molecular imaging modalities, such as positron emission tomography (PET) and single-photon emission computed tomography (SPECT), provide functional insights into breast cancer. These approaches enable non-invasive visualization of tumors and molecular markers, thereby enhancing early detection and improving treatment precision. Furthermore, the development of radionuclide probes targeting specific receptors and biomarkers has greatly advanced breast cancer theranostics [59].

Guzik et al. explored the use of [^177^Lu]Lu-DOTA-folate as an immune stimulus to enhance the efficacy of anti-CTLA-4 immunotherapy in a syngeneic mouse model of breast tumors. Experiments demonstrated specific targeting of NF9006 tumor cells by the radiolabeled folate in vitro and in vivo. Although [^177^Lu]Lu-DOTA-folate or anti-CTLA-4 therapy alone had a minimal impact on tumor growth and survival, which did not significantly enhance survival time in mice compared to control groups, their combination resulted in marked tumor growth inhibition and prolonged survival beyond 70 days without noticeable side effects. This synergistic effect is due to immunogenic cell death being inhibited by [^177^Lu]Lu-DOTA-folate-induced radiation, which makes tumor microenvironments more responsive to immune checkpoint blockade. These results showed the potential of folate-based radioconjugates to enhance immunotherapy strategies and hold promise for further clinical research [60].

Aranda-Lara et al. developed [^177^Lu]Lu-Folate-Bombesin ([^177^Lu]Lu-Folate-BN) for the imaging and therapy of FR and GRPR-positive breast tumors. Preclinical studies on the T47D human breast cancer cell line and T47D tumor-bearing mice, which delivered 23.97 ± 2.1 Gy after 74 MBq injection, with precise Micro-SPECT/CT imaging, demonstrating the promising potential of [^177^Lu]Lu-Folate-BN as a theranostic radiopharmaceutical due to their concomitant identification of GRPR and FRα. The results obtained warrant a further clinical study specifically aimed at evaluating in vivo tumor uptake, quantifying radiation dose delivered, and assessing therapeutic efficacy [61]. Furthermore, in another study, they synthesized the Lys1 (α,γ-Folate)-Lys3 (EDDA/HYNIC)-Bombesin (1–14) conjugate ([^99m^Tc] Tc-Bombesin–Folate) to target FRα and GRPR, and analyzed its potential in vitro and in vivo. The purity of [^99m^Tc]-labeling was 96 ± 2.1%, which was achieved via High-performance liquid chromatography (HPLC), and the preclinical results showed strong uptake (5.43% ID/g), inhibited by cold FA/Bombesin and confirming dual FRα/GRPR targeting for imaging improvement [62].

In addition, Mendoza-Nava et al. performed a preclinical study on the development of a ^177^Lu-labeled dendrimer (PAMAM-G4) conjugated with folate and bombesin, incorporating gold nanoparticles (AuNPs) for the targeted imaging and therapy of breast tumors overexpressing FR and GRPR. Actually, AuNPs were synthesized within the internal cavity and functionalized with folate and bombesin for FR and GRPR co-targeting, also DOTA chelators for dual-receptor targeting and radiolabeling. Comprehensive physicochemical characterization of AuNPs showed enhanced luminescent properties suitable for optical imaging. The formulation achieved high radiochemical purity (>95%) and 41.15% tumor cell uptake, highlighting its potential for dual optical and nuclear imaging not only in breast tumors overexpressing GRPRs and FRs, but also for targeted radiotherapy [63]. In other experiments performed by Gupta et al. they synthesized [^177^Lu] Lu-IONP-Folate for the treatment of folate receptor-positive cancers, aiming to reduce renal radiation exposure. Using SPECT/CT voxel-based dosimetry and results showed a ~50% lower kidney dose than [^177^Lu]Lu-Folate, making it a safer, personalized TRT option for breast cancer, which showed their potential for clinical application when using the voxel-based dosimetry method. Additionally, IONP integration enables improved safety and supports the development of individualized therapeutic planning [64].

**Figure 3:**
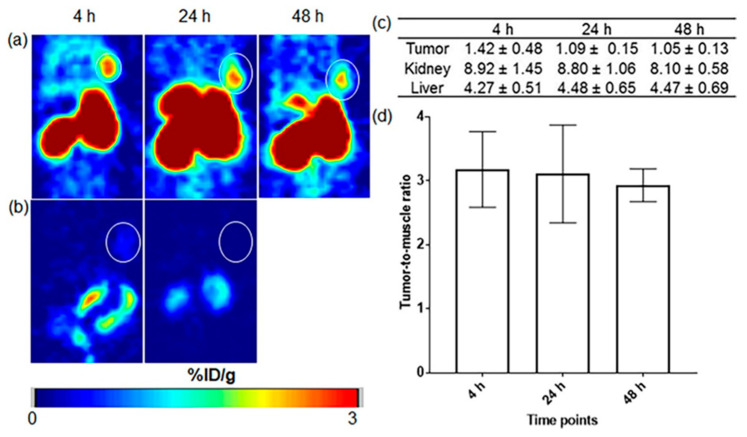
PET images of mice with MDA-MB-453 tumors show (**a**) uptake of ([^89^Zr]Zr-Cbl at 4, 24, and 48 h post-injection (p.i.), with (**b**) reduced tumor uptake when co-injected with excess unlabeled Cbl. Quantitative analysis includes (**c**)%ID/g values for key organs and (**d**) tumor-to-muscle ratios, with tumors which marked by white circles. Reprinted with permission from Ref. [65], Copyright 2017, ACS Omega.

Kuda-Wedagedara et al. reported the synthesis of 89Zirconium-labeled cobalamin ([^89^Zr]Zr-Cbl), which was modified with desferrioxamine and proved successful and suitable for tumor imaging. The studies were conducted in both in vitro and in vivo settings on a positive breast cancer cell line and mice. Additionally, they evaluated the uptake and pharmacokinetics of the CD320-positive MDA-MB-453 breast cancer cells and xenografted mice that confirmed targeting the CD320 receptor which mediates vitamin B_12_ internalization via receptor-mediated endocytosis. However, under sustained therapeutic pressure, may lead to tumor adaptation or altering receptor trafficking, thereby limiting tracer uptake and long-term efficacy. The in vitro test showed a 6–10-fold higher uptake of [^89^Zr]Zr-Cbl rather than uptake in the presence of an excess of cyano-Cbl (CN-Cbl), also PET imaging and biodistribution study in nude mice exhibited a clear visualization of the tumor and high accumulation in kidneys and liver after 48-hr post-injection respectively (Figure 3). The results showed the feasibility of synthesizing a Cbl-based tracer that can be utilized for both in vivo and ex vivo studies of Cbl trafficking. This tracer has the potential to visualize tumors expressing transcobalamin (TC) receptors, such as CD320. Thus, monitoring receptor expression heterogeneity is important to overcome potential resistance mechanisms in transcobalamin receptor–based therapies [65].

Ucar et al. developed folate-targeted nanostructured lipid carriers (NLCs) for delivering paclitaxel (PTX) to folate receptor (FR)-overexpressing tumors. In vitro studies using MCF-7, HeLa, and A549 cell lines, and in vivo rat models, demonstrated enhanced cellular uptake and cytotoxicity in FR-positive cells. The NLCs were modified with folate-PEG-CHEMS to improve targeting and circulation, then radiolabeled with [^99m^Tc(CO)_3_]^+^. Radiolabeling was efficient, and biodistribution studies confirmed higher uptake in FR-positive tissues. These results support [^99m^Tc]Tc-(CO)_3_-PTX-NLCs as promising candidates for FR-targeted theranostic applications, highlighting the potential of radiolabeled lipid-based nanoparticles in precision oncology [66].

The study by Temma et al. focused on radiolabeling anti-MT1-mAb with [^99m^Tc] to detect MT1-MMP, an enzyme with high expression on the surface of malignant tumor cells, which is suitable for a diagnostic target. HYNIC was used as a chelating agent, and the probe synthesis was performed with significant immunoactivity (84%) and high radiochemical purity (>92%). Western blotting confirmed the expression of MT1-MMP in breast tumor models, and the biodistribution study revealed a specific accumulation of the tumor over time, with tumor-to-blood ratios exceeding 1.3 after 48 h. Also, the dose effecting was 5.0 mSv/MBq. The results suggested [^99m^Tc]Tc-anti-MT1-MMP mAb as an imaging agent that has the potential for breast cancer diagnosis in nuclear medicine for future translational imaging clinically [67].

Silindir-Gunay et al. designed liposomal imaging agents for SPECT and SPECT/CT to assess their effectiveness. Folate-conjugated, PEGylated, [^99m^Tc]-radiolabeled liposomes (Folate-PE:DMPC: DTPA-PE) demonstrated prolonged circulation, high tumor uptake, and specific targeting of folate receptors in 4T1 cells. Thus, these liposomes suggested promising alternatives to [^99m^Tc]Tc-Sestamibi, while further in vivo studies are required for validation [68]. Mollarazi et al. focused on developing biocompatible, water-soluble [^153^Sm]-labeled chitosan nanoparticles (NPs) with folate and polyethyleneimine for targeted cancer therapy. The spherical NPs (~250 nm) were stable and exhibited significant uptake in MCF-7 and 4T1 cells with specific FR targeting. In vivo studies exhibited renal excretion and minor tumor accumulation up to 96 h, suggesting that folic acid-functionalized chitosan NPs could be promising for FR-targeted therapy also due to their rapid removal from the kidneys in comparison with other presented liver-accumulating agents [69].

In another study, Guo et al. introduced a new FR-targeting agent for SPECT imaging, focusing on improving contrast through multimerization and PEGylation. [^99m^Tc]-labeled folate derivatives displayed high tumor uptake in KB cells, specific targeting, and significant biodistribution via micro-SPECT/CT imaging. Alongside, the PEGylated monomeric [^99m^Tc]Tc-HYNFA showed the most considerable tumor uptake and imaging contrast, which suggested more effectiveness for designing the radio-folates that could be promising agents for breast cancer diagnosis and treatment monitoring [70].

## 5. Colorectal Cancer-Targeted Vitamin-Based Radionuclide Agents

Colorectal cancer is the second most common cause of cancer-related deaths and the third most common cancer globally. Exposure to risk factors, demographic differences, genetic susceptibility, and genetic mutations impacts its treatment response and prognosis [71]. The development of therapeutic strategies that integrate multiple drugs has gained significant attention in addressing multidrug resistance (MDR). Combination therapies, such as chemotherapy combined with immunotherapy, have demonstrated promising efficacy in targeting key proteins, including the epidermal growth factor receptor (EGFR) and the vascular endothelial growth factor receptor (VEGFR). Furthermore, the identification and validation of critical biomarkers play a crucial role in advancing personalized medicine, enabling a deeper understanding of MDR mechanisms and associated toxicities. These approaches enhance treatment efficacy and also contribute to tailored therapy, leading to improved clinical outcomes [72]. Currently, the increased understanding of radiochemistry, bioengineering, and cancer biology has significantly enhanced the potential of targeted radionuclide therapy to address the efficient treatment of solid tumors [73].

Kotun et al. developed the first PET imaging agent based on a vitamin B12-derivative, [^64^Cu]Cu-B12-en-Bn-NOTA, to assess tumor-associated nutritional demand. Preclinical studies in various human and murine cancer models (HCT116, SKOV-3, B16-F10) showed notable tumor uptake (2.20–4.84% ID/g at 6 h), mediated by transcobalamin II (TCII) receptors. These mechanisms facilitate the cellular internalization of vitamin B_12_ via receptor-mediated endocytosis. Tumor specificity was confirmed by a 95% reduction in uptake following co-injection with native B12. However, high renal accumulation remains a limitation for clinical translation. These findings support vitamin B12-based radiotracers as promising PET probes for imaging tumor metabolism, while emphasizing the need for receptor-expression monitoring with future optimization needed to reduce off-target kidney uptake and validation in higher-species models before clinical application [74].

Above that, the use of folate-conjugated shell-cross-linked nanoparticles (SCKs) for targeted tumor imaging and therapy, highlighted by Rossin et al. illustrated a specific FR interaction and [^64^Cu] radiolabeling for biodistribution studies. The SCKs showed significant RES uptake, long circulation, and passive tumor accumulation. Additionally, folate-specific binding was observed in smaller tumors, indicating the potential of SCKs for early-stage cancer treatment and suggesting a promising approach for targeted imaging and therapy in colorectal cancer [75].

Cheal et al. conducted in vitro and in vivo experiments on colorectal cancer cell lines and mouse models. They introduced a glycodendrimer-based clearing agent (CCAα16-DOTA-Y^3+^) for pre-targeted radioimmunotherapy (DOTA-PRIT) using [^177^Lu] Lu-DOTA-B. The agent effectively delivered radiation to tumors (468 cGy/MBq) and favorable tumor-to-normal tissue dose ratios, with the highest ratio (~550) for the stomach. The radiolabeling with [^177^Lu] ensured precise imaging and therapeutic targeting, indicating strong selectivity and minimal off-target effects. These findings highlight the potential of high-therapeutic index (high-TI) DOTA-PRIT for targeted treatment of stomach tumors, ensuring effective tumor treatment while minimizing the risk of damage to normal stomach tissues [76].

**Figure 4:**
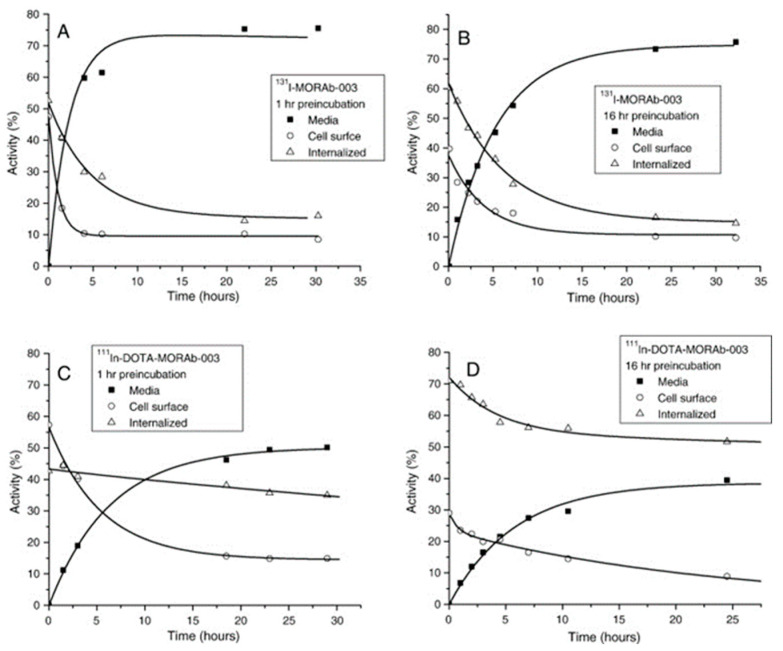
Radioactivity distribution in IGROV1 cells after incubation at 37 °C with either [^131^I]I-MORAb-003 ((**A**): 1 h; (**B**): 16 h) or [^111^In]In-DOTA–MORAb-003 ((**C**): 1 h; (**D**): 16 h). Reprinted with permission from Ref. [77]. Copyright 2008, Elsevier.

In other preclinical studies, Smit. Jones et al. characterized and radiolabeled monoclonal antibody MORAb-003 which targeted folate receptor alpha (FRA). MORAb-003 was conjugated with DOTA and labeled with [^111^In] and [^131^I]. Cellular retention was assessed in human ovarian adenocarcinoma (IGROV1) and colon adenocarcinoma (SW620) cell lines, which demonstrated high FRA affinity and significant internalization, with [^111^In] exhibiting intracellular retention. At the same time, [^131^I] was released as iodide that showed low intercellular retention (Figure 4). In vivo, biodistribution studies in tumor-bearing mice showed that [^111^In]In-DOTA-MORAb-003 had significant tumor uptake (32 ± 5% ID/g at 4 days) and a 110-h blood clearance rate. Organ clearance followed the same pattern, showing low retention after 8 days. In addition, clinical trials in a small group of patients validated effective tumor targeting and favorable biodistribution of MORAb-003, supporting its potential for radioimmunoscintigraphy, radioimmunotherapy, and targeted treatment of FRA-expressing cancers [77].

Kim et al. examined tumor targeting using triphenylphosphonium (TPP) cations (membrane potential-based) and FA (FR-targeting) with FDA-approved silica nanoparticles (SNPs) as carriers. The radiochemical purity of [^89^Zr]-labeled SNPs was >99%. PET imaging and cellular uptake analysis revealed that TPP-modified SNPs (SFT-5) exhibited a significant tumor affinity in CT-26 cancer cells, confirming that targeting membrane potential via TPP is more effective compared to FA receptor-based targeting [78]. The other study, conducted by Kim et al., showed significant potential of micro-scale silica wires (SMWs) as a drug carrier for targeting FR, which was conjugated with FA and labeled with [^89^Zr] for PET imaging in preclinical assays. They confirmed that the FA-conjugated sample stayed at the tumor site for a longer period and reported the unique in vivo behavior of [^89^Zr]Zr-FA-SMWs [79].

Choi et al. conducted a study on synthesizing the HBED-CC-EDBE-folate precursor (HCEF) and labeling it with [^68^Ga], which was assessed by HPLC and TLC regarding radiochemical yield and purity. The study showed efficient radiolabeling with a 98% yield at room temperature. Preclinical studies showed strong binding of the probe in folate receptor-positive cancer cells and selective accumulation in tumors, confirming [^68^Ga]Ga-HCEF as a promising tool for cancer diagnosis using PET imaging [80].

Khatik et al. developed a novel oral drug delivery system for the targeted treatment of colorectal cancer using curcumin-loaded gliadin nanoparticles (Gd NPs), surface-functionalized with folic acid (FA) and coated with Eudragit S-100 (ES) for colon-specific release. This folate-targeted nanocarrier was designed to exploit the overexpression of folate receptors in tumor cells. In vitro studies confirmed its ability to induce apoptosis in cancer cells, while in vivo gamma scintigraphy demonstrated efficient tumor localization and sustained release of the therapeutic agent. The results highlight the potential of this system as a safe and effective strategy for prolonged oral therapy in colorectal cancer [81].

Additionally, [^99m^Tc]Tc-PAMA-cobalamin, a novel radiolabeled vitamin B12 derivative synthesized by Sah et al., targets tumors by avoiding normal tissue uptake through transcobalamin. It binds to a protein expressed in many tumors, like haptocorrin. In a study involving 10 metastatic cancer patients, 6 showed positive tumor uptake in lung, colon, breast, and hypopharyngeal cancers, indicating tumor-specific uptake via haptocorrin-mediated binding. Administering cold cobalamin before scanning enhanced tumor uptake and improved imaging quality while maintaining low renal uptake, that showed long-term dosimetry considerations needed for clinical translation. Overall, this research confirms [^99m^Tc]Tc-PAMA-cobalamin could be a promising tool for cancer imaging [82].

## 6. Brain Cancer-Targeted Vitamin-Based Radionuclide Agents

Brain tumors, or brain cancer, are characterized by the uncontrolled proliferation of abnormal or malignant cells within the brain or its surrounding tissues, leading to the formation of pathological masses. Patients diagnosed with malignant brain tumors frequently face severe clinical challenges, substantial morbidity, and poor prognosis [83], with an average survival of about 14 months with aggressive treatment. A key challenge that needs to be addressed is the ability of these compounds to cross the blood–brain barrier (BBB), which limits the entry of many chemotherapeutic agents into the brain. Despite conventional therapies, receptor-mediated transcytosis remains a problem with limited efficacy and difficulty. As vitamins A and C, for example, are showing promise in this regard, it would be worthwhile to explore how other vitamins or nanoconstructs based on them could be used to enhance the targeting related to BBB crossing [84,85]. However, recent advancements in nanotechnology make an innovation route in cancer therapy [86]. Moreover, progress in nuclear medicine has introduced promising innovative strategies for glioblastoma treatment, particularly through radioimmunotherapy, radiopeptide therapy, and radionanoparticles. Additionally, radiopharmaceuticals have shown the ability to enhance stabilizing or improving neurological conditions with minimal side effects, highlighting their potential as an effective tool for glioblastoma therapy [87].

Miner et al. highlighted [^18^F]F-FOL as a folate-based PET imaging agent that targets overexpressed folate receptors (FRs) for detecting gliomas in a rat model and assesses FR expression in human glioblastoma samples. PET imaging of glioma-bearing rats demonstrated a significantly higher tumor-to-brain uptake ratio (TBR) over time for [^18^F]F-FOL compared to [^18^F]F-FDG. Ex vivo studies confirmed the superior TBR of [^18^F]F-FOL, supported by immunostaining, which revealed upregulated FR-α in glioma regions and FR-β at the tumor periphery. Moreover, in human glioblastoma samples, similar FR expression patterns were observed. These results suggest the potential of FR-targeted agents like [^18^F]F-FOL for the detection and treatment of gliomas in both preclinical and clinical settings [88].

In addition, Liang et al. introduced [^18^F]F-AlF-NOTA-Asp2-PEG2-Folate, a PET tracer designed with a hydrophilic linker to improve pharmacokinetics. The tracer was created efficiently within 30 min, and FR in KB cells showed high specificity and affinity. In KB tumor-bearing mice, [^18^F]F-AlF-NOTA-Asp2-PEG2-Folate indicated good tumor uptake (1.7% ID/g), reduced accumulation in the kidney and liver, and rapid clearance from normal tissues in comparison with [^18^F]F-AlF-NOTA-Folate. The folate receptor via high-affinity endocytosis facilitates ligand internalization which make selective tumor accumulation of folate-conjugated tracers. However, variable FR expression and disruptions in receptor transport or recycling can diminish tracer accumulation, particularly in more aggressive or resistant tumors. Thus, these findings hold the [^18^F]F-AlF-NOTA-Asp2-PEG2-Folate, a promising option for PET imaging in FR-positive tumors and a critical improvement for clinical translation [89].

Shi et al. performed an experiment using a folate-receptor-targeted imaging probe, [^64^Cu]Cu-DOTA-FA-ICG, NIR-II fluorescence imaging of glioblastoma (GBM). The probe formed nanoparticles and accumulated specifically in GBM tumors, which enabled real-time tumor visualization during surgery. Nonetheless, intratumoral heterogeneity and microenvironment-driven modulation of FR levels may limit complete tumor delineation and contribute to residual disease, so safe translation of DOTA-FA-ICG and ^64^Cu-DOTA-FA-ICG is crucial. This innovation improves tumor differentiation and surgical accuracy [90].

Korany et al. developed a direct synthesis method for amorphous selenium nanoparticles (SeNPs) coated with vitamin C and radiolabeled with [^99m^Tc] for in vivo imaging studies in both healthy and tumor-bearing mice. The resulting nano complex demonstrated optimal particle size, high colloidal stability, and a radiolabeling yield of 96 ± 2%, maintaining stability for over six hours. Biodistribution analysis revealed favorable target-to-non-target ratios in tumor-bearing models. While vitamin C was primarily employed as a stabilizing and coating agent, its presence may also contribute indirectly to the radiolabeling efficiency and biocompatibility of the system. These findings support the potential of [^99m^Tc]Tc-VitC-SeNPs as promising agents for solid tumor imaging [91].

A radiolabeled folate-loaded micelles or non-invasive intranasal delivery, developed by Upadhaya et al., to target brain tumors by exploiting folate receptor overexpression in glioma cells, the micelles were efficiently labeled with [^99m^Tc] (>95% yield). Their nanoscale size and mucoadhesive properties facilitated direct brain delivery. In vivo studies showed high brain uptake, and SPECT imaging confirmed effective targeting, highlighting the potential for diagnosing brain and other cancers [92].

Nosrati et al. introduced [^99m^Tc]Tc-BPTG-1 and [^99m^Tc]Tc-BPTG-2 for glioblastoma imaging as potential radiotracers. In vitro tests showed strong uptake in U87-MG cells, with tumor accumulation exceeding muscle and brain levels in mice. The radiotracers were cleared via the liver and kidneys, which showed the greatest activity, highlighting their potential for GBM diagnosis [93].

Another radiopharmaceutical targeted the folate receptor (Ga-DF-Folate) was developed by Mathias and colleagues using [^66^Ga] and [^68^Ga] radionuclides for PET imaging. In mice with folate-receptor-positive tumors, [^66^Ga] imaging was successful despite its high positron energy. Dual-isotope autoradiography with [^18^F]F-FDG and [^111^In]In-DTPA-Folate revealed tumor heterogeneity and regional tracer variations. Given the prominent role of kidneys in folate tracer clearance, precise renal dosimetry remains a major consideration for implementing FR-targeted Ga-labeled agents in clinical imaging [94].

In addition, Zhang et al. designed a novel [^68^Ga]-labeled PET tracer targeting FRs, such as Pteroyl-Lys-DOTA and Pteroyl-Lys-DAV-DOTA. These compounds were successfully synthesized, radiolabeled with [^68^Ga], and evaluated in FR-positive KB cells and tumor-bearing mice. Both showed high tumor specificity, with tumor uptake values of 10.06% ID/g and 11.05% ID/g at 2 h post-injection and clear tumor visualization in Micro-PET imaging, which supported their potential as FR-targeted PET probes for applications in cancer diagnosis and therapy [95].

## 7. Prostate Cancer-Targeted Vitamin-Based Radionuclide Agents

Prostate cancer represents a major global health challenge and is among the leading causes of cancer-related mortality in men. A substantial proportion of patients are diagnosed with advanced-stage disease. Clinical diagnosis primarily relies on the digital rectal examination and measurement of prostate-specific antigen (PSA) levels, with confirmation obtained through histopathological analysis of biopsy tissue. Current therapeutic interventions for localized disease include radical prostatectomy and radiotherapy, while advanced or metastatic disease is managed with systemic therapies such as androgen deprivation therapy and chemotherapy. To overcome challenges including drug resistance and dose-limiting toxicities, emerging strategies such as combination therapies and nanoparticle-based drug delivery systems are under investigation to improve therapeutic efficacy and reduce adverse effects [96].

Ak et al. introduced [^99m^Tc]Tc-folate–PEG–doxorubicin, a radiolabeled nanoconjugate for imaging prostate cancer and localizing the drug. Based on biodistribution studies performed in rats, folate ligands have a higher affinity for folate receptors than control groups. With a high specificity for tumor tissue, this radiolabeled conjugate suggests strong potential for prostate cancer diagnosis and real-time tracking of drug distribution [97]. Also, L. Dhas et al. created folic acid-conjugated chitosan-coated PLGA nanoparticles for targeted prostate cancer therapy. Bicalutamide-loaded NPs demonstrated efficient drug encapsulation, controlled release, and in vitro cytotoxicity in DU145 human prostate cancer cell lines. They exhibited higher cytotoxic effects compared to the uncoated nanoparticles (UPN) and the pure drug. Stability and hemolysis studies validated its safety and potential for prostate cancer treatment with further in vivo investigations [98].

A new peptide derivative of folic acid known as EC20, identified by P Leamon et al., to coordinate with [^99m^Tc] for potential cancer imaging applications. EC20 illustrated a high affinity for FR-positive tumor cells. In vitro study confirmed that EC20 could compete effectively with 3H-folic acid for cell binding, either alone or as a metal chelate. Upon intravenous injection into Balb/c mice, [^99m^Tc]Tc-EC20 exhibited rapid clearance from circulation (plasma half-life ~4 min) and was excreted via urine. [^99m^Tc]Tc-EC20 predominantly accumulated in FR-positive tumors and kidneys, were confirmed by Gamma scintigraphy and biodistribution studies. These results suggest a significant potential of [^99m^Tc]Tc-EC20 as a non-invasive imaging agent for detecting FR-positive human cancers [99].

Guo et al. designed [^99m^Tc]Tc-HYNIC-D1-FA2, a radiolabeled dimeric folate-based tracer, for targeted imaging of FR-overexpressing tumors. The tracer was synthesized via click chemistry and exhibited strong tumor binding (IC50 = 19.06 nM) and high hydrophilicity (log P = −2.52). In KB tumor-bearing mice, a biodistribution study demonstrated high tumor and kidney uptake, which was notably reduced when free folic acid was used for blocking, as observed upon pre-saturation with excess folic acid, confirming high receptor specificity of the ^99m^Tc-labeled folate derivatives. Micro-SPECT/CT imaging showed effective tumor visualization, suggesting that [^99m^Tc]Tc-HYNIC-D1-FA2 could be a promising FR-targeted imaging agent for cancer treatment [100].

In addition, two folate derivatives, CN5FA and CNPFA, were synthesized by Feng et al. and radiolabeled with [^99m^Tc] to create [^99m^Tc]Tc-CN5FA and [^99m^Tc]Tc-CNPFA complexes. These complexes demonstrated high radiochemical purity (>95%), hydrophilicity, and good in vitro stability. Competitive binding assays in KB cells confirmed the specificity of this receptor for folate. In vivo, studies in KB tumor-bearing mice revealed selective tumor uptake, with [^99m^Tc]Tc-CNPFA exhibiting higher tumor-to-muscle ratios than [^99m^Tc]Tc-CN5FA due to its superior SPECT/CT imaging. These findings indicate that the [^99m^Tc]-labeled complexes could be promising agents for prostate cancer imaging [101].

Ross et al. developed a PET imaging tracer targeting FR, [^18^F]F-click folate, for tumors that utilize click chemistry. They synthesized in high radiochemical yield and strong binding FR affinity with the 25–35% and (Ki = 9.76 nM), respectively, in in vitro study. PET imaging in mice demonstrated significant tumor uptake (3.13% ID/g), but high liver excretion resulted in a background signal. While exhibiting good in vitro binding for FR-positive tumors, further optimization is necessary to enhance in vivo imaging quality [102].

Yigit et al. used a radiolabeled form of ascorbic acid with [^99m^Tc] and evaluated its radiopharmaceutical potential in vivo in rats. They successfully labeled the [^99m^Tc] ascorbic acid ([^99m^Tc]Tc-AA) using the stannous chloride method, achieving a 93% yield under optimal conditions. After injection into Wistar rats, a biodistribution study showed the [^99m^Tc] Tc-AA accumulated in the prostate and kidneys at 60 min. Given the prominent kidney accumulation, renal dosimetry is a key consideration should ^99m^Tc-AA advance, which is suggested as a promising imaging agent for these organs in future diagnostic applications [103].

Schibli et al. developed a novel [^99m^Tc] radiolabeled vitamin B12 derivative (PAMA-4-B12) to enhance tumor targeting and diagnostic imaging while minimizing non-specific binding to transport proteins, such as TC, which often leads to unwanted accumulation in the liver, kidneys, and glands. Tumor localization occurs through vitamin B_12_ receptor–associated endocytosis. The radiochemical purity of PAMA-4-B12 was >95%, and targeted tumors efficiently in various animal models, including prostate carcinoma, showed fast renal elimination and minimal liver uptake. In a clinical pilot study with patients with different cancers, including prostate carcinoma, PAMA-4-B12 showed fast renal elimination, but there was unexpectedly high liver uptake indicating species-dependent pharmacokinetics. These findings suggest that PAMA-4-B12 holds promise as a diagnostic and therapeutic agent for certain carcinomas, with ongoing investigations needed to address liver uptake, especially for clinical prostate cancer applications [104].

Waibel et al. investigated modified vitamin B12 derivatives for targeted imaging of hyperproliferative cancer cells. Using colon (LS 147T) and prostate (LNCaP) carcinoma cell lines, as well as in vivo mouse models, they assessed biodistribution and tumor specificity. By altering B12 to bypass the transcobalamin II pathway, off-target uptake in normal tissues was minimized and enhancing tumor accumulation. Transcobalamin I was proposed as a tumor-specific receptor. TCI-mediated endocytosis enhanced tumor uptake and improved tumor-to-blood ratios while reducing renal and hepatic exposure, which highlighting the clinical potential of B12-based radiopharmaceuticals with consideration to heterogeneity in TCI expression across tumor types that warrants further investigation [105].

## 8. Ovarian Cancer-Targeted Vitamin-Based Radionuclide Agents

Ovarian cancer (OC) is a heterogeneous disease with multiple subtypes, each requiring different treatment approaches. Due to the lack of effective screening, it is often diagnosed late, and standard treatments such as surgery and platinum-based chemotherapy are performed. High-grade serous carcinoma (HGSC), the most prevalent type, initially responds well to treatment but often develops resistance to treatment [106]. For this reason, early detection of cancer is crucial for effective treatment and better survival outcomes. Nuclear medicine plays a significant role in cancer diagnosis and therapy. Advancing SPECT and PET-based theranostic tools for ovarian cancer highlights their advantages for diagnosis and treatment [107,108,109].

Reber et al. designed a radioiodinated folate conjugate to selectively target FR-positive tumors while minimizing kidney toxicity. This addressed the issue of high kidney uptake as a key limitation of traditional radiofolates, which created a risk of renal radiation damage during therapy. Two variants, [^125^I]I-2 and [^125/131^I]I-4, showed high stability, FR-specific binding and strong tumor accumulation. In vitro studies displayed [^131^I]I-4 reduced FR-positive tumor cell viability. Pre-injection with pemetrexed improved tumor imaging via SPECT/CT, suggesting these conjugates are promising for the diagnosis and therapy of FR-positive cancers like ovarian cancers [110].

Moreover, Aljammaz et al. developed [^124^I]I-SIB-folate and [^124^I]I-SIP-folate conjugates for enhancing the imaging of folate receptor-positive cancers. They employed a rapid two-step process, achieving high radiochemical yields (>90% and >60%) and purity (>98%). In vitro and in vivo studies demonstrated effective tumor targeting, particularly for [^124^I]I-SIP-folate, with good biodistribution and receptor-specific uptake. All the results showed the potential of [^124^I]I-SIP-folate as a PET imaging prob for ovarian cancer diagnosis, staging, and treatment monitoring [111].

Aljammaz et al. synthesized novel [^18^F]F-fluorobenzene and pyridinecarbohydrazide-folate/methotrexate conjugates and used nucleophilic displacement reactions. They achieved high radiochemical yields and purity with (>80%) and (>97%), respectively, without requiring HPLC purification, allowing for quick and efficient methods for folate derivative radiofluorination. Various experiments confirmed significant receptor-mediated uptake, while animal experiments demonstrated fast blood clearance and significant tumor accumulation, particularly for [^18^F]F-2-folate. Micro-PET imaging validated these results, highlighting [^18^F]F-2-folate could be a promising molecular probe for detecting and monitoring folate receptor-positive cancers, such as ovarian cancer and its metastases [112]. In another experiment, Aljammaz et al. improved PET Imaging of folate receptor-positive tumors by developing [^18^F]F-FDG-folate and methotrexate conjugates ([^18^F]F-5 and [^18^F]F-8). These were produced rapidly and in a one-step process with high yields (>80%) and purities (>98%). In vivo studies in mice showed rapid clearance and significant tumor uptake, particularly for [^18^F]F-5. Receptor-blocking experiments via excess folic acid confirm specific FR-mediated internalization via high-affinity endocytosis. While, receptor overexpression favors tumor targeting, resistance may occur through downregulation of FR or altered folate transport pathways but suggested [^18^F]F-5, a potential imaging probe for ovarian and other cancers [113].

Siwowska et al. evaluated [^47^Sc]Sc-labeled DOTA-folate as a potential for targeted radionuclide therapy in folate receptor-positive ovarian cancer, compared to [^177^Lu]Lu-folate and [^90^Y]Y-folate. In vitro studies showed effective reduction in tumor cell viability in [^47^Sc]Sc-folate, which was the same for [^177^Lu]Lu-folate, while the [^90^Y]Y-folate, due to higher energy emission, was more potent. In vivo experiments in mice demonstrated comparable tumor growth inhibition and prolonged survival (39–43 days vs. 26 days in controls) with no severe side effects for all three radiofolates. Therefore, the results indicate [^47^Sc] as a promising alternative to [^177^Lu] and a promising candidate for clinical use and theranostic applications [114].

Guo et al. investigated a novel therapeutic approach for ovarian cancer by developing [^177^Lu]Lu-FA-DOTA-PEG-PLGA nanoparticles. These nanoparticles integrate FR-mediated targeting, PEG-PLGA for biodegradability and DOTA for radiolabeling, achieving high labeling efficiency (97–98%) and, minimal kidney accumulation. Micro-SPECT/CT imaging in OC tumor-bearing mice confirmed tumor localization, and treatment significantly inhibited tumor growth and, reduced ascitic fluid also, no major toxicity was shown through histological analysis. These findings suggest that [^177^Lu]Lu-FA-DOTA-PEG-PLGA nanoparticles could serve as a promising strategy for OC treatment [115].

To enhance DDS by overcoming challenges related to safety, efficacy, and tumor penetration, Wu et al. developed EC112002, an ultrasmall FRα-targeted silica nanoparticle drug conjugate (CDC); this nanoplatforms demonstrated high specificity for FRα, efficient tumor penetration, rapid systemic clearance, and minimal off-target effects following an extensive screening of over 500 formulations. In preclinical studies comparing EC112002 with traditional antibody–drug conjugates (ADCs), EC112002 showed enhanced tumor penetration into 3D tumor models and significant targeted cytotoxicity. These attributes position EC112002 as a promising clinically translatable DDS with potential applications in targeted cancer therapies, including ovarian cancer [116].

Tang and Chen et al., explored a folate-targeted albumin nanoparticle system, specifically [^188^Re]Re-folate-CDDP/HAS MNP, for evaluating the therapeutic efficacy in ovarian cancer treatment. Preclinical studies were assessed in SKOV3 cells and tumor-bearing mice using various combinations of chemotherapy, radiotherapy, and hyperthermia. The therapeutic mechanism induced DNA damage and folate receptor–mediated targeting of tumor cells by delivering rhenium-188 for β− radiation, cisplatin enhances cytotoxicity through DNA cross-linking and magnetic hyperthermia to enhance radiosensitivity and drug penetration for synergistic antitumor effects. All treatments groups included (B) chemotherapy; (C) radiotherapy; (D) hyperthermia; (E) chemotherapy and radiotherapy; (F) chemotherapy and hyperthermia; (G) radiotherapy and hyperthermia; (H) chemotherapy, radiotherapy and hyperthermia, significantly suppressed tumor growth and the strongest effect achieved by triple combination (H) with (*p* < 0.05). Apoptosis rates increased across groups, reaching 57.16% in group H. In vivo studies further confirmed a significant reduction in tumor mass among treated groups, specifically in group H (*p* < 0.05). Thus, all findings highlight the effect of multimodal therapy for ovarian cancer [117].

A novel system presented by Oumzil et al. named [^99m^Tc]Tc-labeled cisplatin nanoparticles ([^99m^Tc]Tc-NP-PEGFA), is functionalized with folic acid for targeted drug delivery. The researchers synthesized PEG-based nucleolipids for stable nanoparticle formation and efficient radiolabeling with [^99m^Tc]. In vitro studies of these NPs on two ovarian cancer cell lines (IGROV1 and SKOV3) showed high stability and cellular uptake, whereas prolonged blood circulation and enhanced tumor accumulation were observed in vivo, supporting their potential as targeted drug delivery systems in theranostic applications for cancer therapy [118].

## 9. Meta-Analysis of Tumor Uptake Ratios

A comparative analysis of the tumor uptake data, expressed as percentage injected dose per gram (% ID/g) from the preclinical studies reviewed, reveals distinct patterns in the targeting efficacy of different vitamin-based radiopharmaceuticals across various cancer types. This meta-analysis highlights the most promising agents and identifies trends in performance.

The compiled data, primarily from Table 1 and Table 2, and in-text results, indicate that folate-based radioconjugates consistently demonstrate high and specific tumor accumulation. For instance, folate-targeted agents showed a wide range of tumor uptake, from moderate levels of ~1.7–5.4% ID/g to exceptionally high values exceeding 10–17% ID/g in optimized systems. Notably, Muller et al. reported a remarkable 17.56% ID/g at 4 h for the albumin-binding folate conjugate [44]. Similarly, Zhang et al. demonstrated high uptake with [^68^Ga] Ga−Pteroyl−Lys conjugates, achieving 10.06% ID/g and 11.05% ID/g [95]. These high values are often attributed to strategies that enhance circulation time, such as albumin binders or PEGylation, which promote the Enhanced Permeability and Retention (EPR) effect and improve receptor-mediated uptake.

In contrast, vitamin B12-based agents generally exhibited more moderate but still significant tumor uptake. The PET tracer [^64^Cu] Cu−B12−en−Bn−NOTA showed uptake in the range of 2.20–4.84% ID/g. A key challenge for B12 derivatives has been high renal accumulation, as seen with.

When comparing radionuclides, diagnostic pairs provided critical insights. For example, studies using the theranostic pair ^68^Ga (for PET) and ^177^Lu (for therapy) for the same folate vector often showed correlative high uptake, validating the theranostic principle. The tumor uptake ratios between these isotopes were generally consistent within the same targeting platform, supporting the use of ^68^Ga imaging for predicting ^177^Lu therapy efficacy.

A cross-cancer comparison further elucidates the versatility of these agents. The highest tumor uptake values were frequently reported in KB (nasopharyngeal) and ovarian cancer models, which are known for their high folate receptor (FR) expression. For example, folate conjugates in FR-positive KB tumors consistently yielded uptake values above 5% ID/g, with many studies reporting >10% ID/g [44,95]. This contrasts with some gastric and brain cancer models, where initial folate tracers showed lower or more variable uptake (e.g., ~1.7% ID/g in brain [89]), potentially due to differences in FR density, vascularization, or the blood–brain barrier.

Statistical trends from the compiled data (with significance often reported as *p* < 0.05 or higher) confirm that receptor specificity is a major driver of uptake. Blocking experiments with excess cold folate or B12 consistently resulted in a statistically significant reduction (often >50–95%) in tumor accumulation, underscoring the receptor-mediated mechanism.

Conclusion of Meta-Analysis: This quantitative synthesis underscores that folate-based radiopharmaceuticals, particularly those optimized with pharmacokinetic modifiers like PEG or albumin-binding motifs, achieve the highest tumor uptake ratios among vitamin-based agents. While vitamin B12 derivatives show promise, their clinical translation may require further engineering to improve tumor-to-kidney ratios. The consistent performance of the ^68^Ga/^177^Lu-folate pair across studies solidifies its role as a leading candidate for theranostic applications in FR-positive cancers. Future research should focus on standardizing uptake reporting and conducting direct, comparative studies of different vitamin ligands within the same model system to definitively rank their efficacy.

## 10. Comparison Between Vitamin-Based and Conventional Radiopharmaceuticals

The development of radiopharmaceuticals has evolved from conventional agents targeting specific pathways to innovative vitamin-based systems that exploit the metabolic dependencies of cancer cells. Table 1 below summarizes the key differences, advantages, and challenges between these two approaches.

## 11. Discussion

This review synthesizes a substantial body of preclinical (from in vitro to in vivo) evidence demonstrating the significant promise of vitamin-based radiopharmaceuticals and nanosystems within the evolving paradigm of precision oncology and theranostics. The rationale behind this approach lies in the elegant exploitation of endogenous biological pathways; vitamins such as folate (B9) and cobalamin (B12) serve as ideal targeting ligands due to their high biocompatibility, low immunogenic potential, and the frequent overexpression of their corresponding receptors on a wide spectrum of tumor cells. The experimental pathway, from the in vitro conjugation and radiolabeling of vitamins to their validation in animal models, consistently demonstrates that these constructs, including prominent examples like [^68^Ga]Ga-DOTA-folate and [^177^Lu]Lu-DOTA-folate, exhibit exceptional tumor specificity. This enables not only high-contrast molecular imaging via PET and SPECT but also targeted radionuclide therapy, thereby localizing cytotoxic effects to malignant tissue while largely sparing healthy surroundings. Furthermore, integration of these vitamins into advanced nanosystems further augments their potential by improving pharmacokinetics, prolonging circulation, and enhancing tumor accumulation through both passive targeting and active receptor-mediated uptake.

A critical comparative analysis of the data reveals distinct profiles for different vitamin-based strategies. Folate-based systems currently represent the most advanced and extensively studied class, often achieving remarkably high tumor uptake values, sometimes exceeding 17% ID/g, though they are frequently challenged by significant renal accumulation, an issue that has been creatively mitigated through the use of albumin-binding entities and PEG linkers. In contrast, vitamin B12 derivatives typically exhibit more moderate tumor uptake and face a different primary hurdle: off-target accumulation in the liver and kidneys due to binding with endogenous transport proteins, a challenge that newer derivatives are being engineered to bypass. When compared to conventional, clinically established radiopharmaceuticals such as [^177^Lu]Lu-PSMA-617, vitamin-based agents offer distinct advantages in terms of low cost and inherent biocompatibility. However, the conventional agents currently hold a superior position in terms of proven clinical efficacy and standardized use for specific indications. This positions vitamin-based theranostics not as a replacement, but as a complementary arsenal, particularly valuable for targeting tumors that lack conventional biomarkers but overexpress vitamin receptors.

Looking forward, a critical evaluation of the field highlights several promising yet underexplored avenues. The research focus has been disproportionately placed on folate and B12, leaving the substantial potential of other vitamins, such as the radioprotective Vitamin E or the receptor-targeting Vitamin A, largely untapped. Furthermore, the true power of these agents may lie in their synergistic potential with other treatment modalities. Preclinical studies, such as those combining [^177^Lu]Lu-DOTA-folate with immunotherapy, demonstrate a powerful capacity to modulate the tumor microenvironment and enhance overall antitumor efficacy, paving the way for novel combination regimens. Some strategies, such as nanoparticles, antibody–drug conjugates, and glutathione-sensing systems, demonstrate complementary roles in theranostics, with each offering distinct advantages in diagnosis, treatment, or both. Several radiolabeled antibodies, including those with [^99m^Tc] and [^177^Lu], have successfully localized tumors and exerted potent cytotoxic effects. In particular, [^99m^Tc] is successfully used for diagnostic purposes in tumor localization in SPECT; similarly, although [^177^Lu] has been applied as a therapeutic radionuclide, demonstrating potent cytotoxic effects on tumors, it can also be monitored during regression through SPECT imaging, allowing for precise detection and monitoring which shows how these two radionuclides are often used in parallel in in vitro experiments. The FDA has approved several well-established radiopharmaceutical agents for use in oncology, including [^177^Lu]lu-PSMA-617, [^177^Lu]lu-DOTATATE, [^131^I]I-iobenguane (MIBG), 223Ra-dichloride, [^90^Y]Y-ibritumomab tiuxetan, and [^153^Sm]Sm-EDTMP [131].

**Figure 5:**
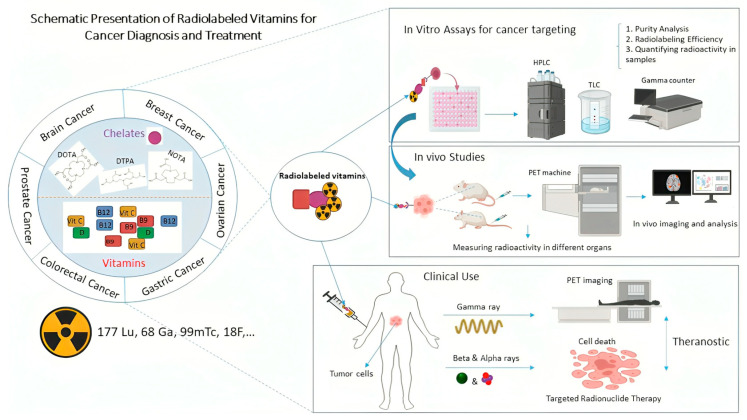
An illustration of the experimental workflow, from in vitro experiments involving vitamins to clinical applications. This schematic presents a theranostic approach to radiolabeled vitamins in various cancers. Radiopharmaceuticals bind to cancer cells selectively, enabling accurate PET/SPECT imaging and targeted radionuclide therapy. In vitro, assays ensure radiolabeling efficiency, while in vivo studies analyze tumor specificity. In clinical studies, gamma emissions aid imaging, whereas beta and alpha particles induce the cancer cells’ apoptosis, enhancing personalized cancer treatment approaches.

This integrated perspective demonstrates the utility of endogenous biomolecules, such as vitamins, for advanced therapeutic applications. Owing to their good biocompatibility, low immunogenic profile, and well-defined metabolic roles, vitamins serve as exemplary molecular scaffolds for the development of targeted radiopharmaceuticals. The experimental pathway, culminating in these applications, is delineated in Figure 5. This schematic summarizes the workflow from in vitro assessment of vitamin conjugates through to pre-clinical validation and potential clinical translation, specifying the key analytical methodologies employed at each stage of the investigative process.

Despite this promising trajectory, the clinical translation of these innovative systems is contingent upon a clear-eyed acknowledgment of current limitations. A primary constraint is the overwhelming reliance on preclinical data; in vitro and small-animal models cannot fully capture the complexity of human tumors and their microenvironments, therefore a robust clinical evaluation is required. The field is also hampered by a lack of standardization in study designs and reporting, making direct comparisons between different agents challenging. Many studies, while demonstrating excellent tumor targeting, lack comprehensive long-term biodistribution and toxicity profiles, especially concerning chronic radiation effects on critical organs like the kidneys. Finally, significant translational barriers persist, including the challenges of scaling up the synthesis of complex nanoconstructs under GMP conditions, navigating regulatory pathways for combination products, and the high costs associated with clinical trials for radiotherapeutics.

Additionally, consistent with precision oncology principles, therapeutic resistance may arise from heterogeneous FR expression, altered receptor recycling, microenvironment-induced modulation, or compensatory folate transport pathways, all of which can reduce the efficacy of FR-targeted imaging or therapy. Integrating these biological, pharmacokinetic, and dosimetric considerations provides a more critical and clinically realistic framework for advancing FR-targeted theranostics. Selecting an appropriate starting dose is important to ensure patient safety, to enable efficiency in reaching the therapeutically relevant dose range [132], and to use data from animal model species and human equivalent models to establish safe starting dosages for first-in-human clinical trials [133]. For instance, the outcome of the therapeutic dose administered is shown in these studies [134,135]. Most studies have employed folate modification to target folate receptor alpha (FRα), which is considered the most therapeutically relevant folate receptor isoform in solid tumors. Also, FRα is preferentially overexpressed in chromosomal instability (CIN)-type gastric cancer, that represented in the majority of the GC studies we mentioned. Although heterogeneity exists between FRα and folate receptor beta (FRβ), most studies have demonstrated that folate-modified agents preferably accumulate in tumors via FRα-mediated endocytosis. Nevertheless, FRα expression may decrease adaptively in response to repeated ligand exposure or therapeutic pressure, potentially reducing tracer accumulation and therapeutic efficacy. Recognizing this receptor plasticity is crucial for optimizing folate-based theranostics and for the development of effective targeting strategies.

Recent advances in nanotechnology have demonstrated considerable potential for developing greener and more cost-effective large-scale technologies, including environmentally sustainable biological processes such as green synthesis. These [136,137] innovations enhance the precision of therapeutic interventions and enable the creation of multifunctional delivery platforms that integrate diagnostic imaging with therapeutic modalities, offering substantial promise for the development of more effective treatment strategies. Thus, future research should focus on the development of advanced molecular designs, exploring innovative biomarkers, and the integration of these therapies with existing modalities, such as immunotherapy, to achieve synergistic outcomes. Realizing the potential of theranostic radionuclides for personalized oncology will be crucial. Further, radioimmunotherapy [138], which combines radiotherapy with immunotherapy, is one of the most advanced treatments, even for the most aggressive forms of cancer, including those resistant to conventional therapies. The support of new Artificial Intelligence tools encompasses an entire treatment process that can be optimized through machine learning [139,140], particularly deep learning algorithms [141]. These technologies can improve dosimetry and treatment response assessment (from in vitro to in vivo), thereby enhancing overall efficiency and personalization in the medical oncology field [142,143], especially in molecular imaging and TRT. The experimental evidence discussed in the manuscript is summarized in Table 2.

## 12. Conclusions

Providing exceptional selectivity for both imaging and therapeutic applications, radionuclides play a crucial role in precision medicine. Depending on the radionuclide used, these agents enable targeted imaging and destruction of tumor cells while minimizing damage to surrounding healthy tissues. In this context, vitamin-based radiopharmaceuticals and nanosystems have emerged as an innovative class of agents in oncology and theranostics. Their unique molecular design combines the selective targeting ability of vitamins with the functional versatility of nanomaterials, allowing for improved diagnostic sensitivity and enhanced therapeutic efficacy. Ongoing advancements in radionuclide attachment methods, radiolabeling efficiency, and molecular design continue to strengthen their potential as dual diagnostic and therapeutic platforms, while also aiming to reduce systemic toxicity and improve biocompatibility.

Comparatively, vitamin-functionalized nanosystems offer several advantages over conventional radiopharmaceuticals. Incorporating vitamin ligands, such as folate, facilitates receptor-specific targeting, enhanced cellular uptake, and prolonged circulation. Due to its strong affinity for folate receptors, which are overexpressed in a wide range of malignancies, folate has been studied most extensively among these compounds. Comparing folate-conjugated nanosystems with non-targeted radiolabeled systems, folate-conjugated nanosystems demonstrate superior tumor localization and reduced non-specific biodistribution. However, while folate-based targeting has achieved significant progress in preclinical settings, its clinical translation faces challenges related to heterogeneity in receptor expression and potential interference from endogenous folate. Other vitamins, including vitamins A, C, D, and E, although less investigated, show promise for their radioprotective and antioxidant properties. Vitamin E [144], in particular, has demonstrated the ability to mitigate oxidative stress and radiation-induced tissue injury, suggesting its potential utility as an adjuvant for improving radionuclide therapeutic efficacy.

A critical evaluation of existing studies reveals that, despite substantial progress, significant challenges remain in the optimization and translation of vitamin-based radiopharmaceuticals. One major limitation lies in the stability of radionuclide conjugations with nanocarriers, as unstable linkages can lead to premature release, suboptimal targeting, and unintended systemic exposure. Moreover, radiolabeling efficiency continues to play a key role in both diagnostic accuracy and therapeutic effectiveness. The majority of the available data are derived from in vitro experiments or small-animal models, providing limited insight into pharmacokinetics, dosimetry, long-term safety, and immunogenicity for humans. Furthermore, vitamins play a dual role in radioprotection and radiosensitization. While antioxidant vitamins such as C and E may protect healthy tissues from oxidative damage, if they are not precisely targeted or controlled, they may also attenuate the cytotoxic effects of radionuclide therapy. Therefore, the development of smart nanosystems capable of spatiotemporal regulation of vitamin release within the tumor microenvironment is essential to maximize therapeutic benefits without compromising tumoricidal efficacy.

Vitamin-based radiopharmaceuticals and nanosystems possess several promising biological and therapeutic properties, but their cost-effectiveness is one of the most important considerations for their widespread adoption and clinical translation. Traditional radiopharmaceuticals often involve complex synthesis procedures, expensive targeting ligands such as monoclonal antibodies, and costly radionuclide production processes, which collectively increase overall treatment expense. In contrast, vitamins offer a more economical and readily available class of targeting molecules due to their natural abundance, low molecular weight, and ease of chemical modification. This intrinsic affordability may substantially reduce production costs, especially when integrated into scalable nanoparticle synthesis methods. Furthermore, vitamin-functionalized nanosystems have the potential to enhance therapeutic precision, thereby decreasing the need for repeated imaging or treatment cycles, which in turn lowers healthcare costs associated with extended hospital stays and adverse effects management. However, comprehensive cost–benefit analyses remain limited, and economic evaluations comparing vitamin-based nanosystems with conventional radiopharmaceuticals are necessary. Future studies should incorporate pharmacoeconomic modeling to assess the long-term financial sustainability of these systems in clinical oncology, balancing innovation with affordability to ensure equitable patient access to advanced radiotheranostic therapies.

Consequently, vitamin-based radiopharmaceuticals and nanosystems hold significant potential to revolutionize precision oncology and theranostics. With continued innovation in molecular engineering, radionuclide conjugation chemistry, and targeted delivery strategies, these systems offer the potential to improve therapeutic selectivity, minimize systemic toxicity, and enable personalized treatment approaches. Despite the widespread use of folate-based nanosystems, exploring other vitamin ligands with radiosensitizing and radioprotective properties could lead to the development of next-generation radiotheranostic technology. Ultimately, the integration of vitamin-functionalized nanosystems into radionuclide therapy may provide a novel approach to cancer treatment, bridging the gap between molecular imaging, targeted therapy, and patient-centered precision medicine.

## Figures and Tables

**Table 1:** Comparison of Vitamin-Based and Conventional Radiopharmaceuticals.

Aspect	Vitamin-Based Radiopharmaceuticals	Conventional Radiopharmaceuticals
Targeting Mechanism	Exploits overexpression of vitamin receptors (e.g., Folate Receptor α, Transcobalamin receptor) or vitamin transport systems on tumor cells [65].	Targets specific biomarkers like PSMA, Somatostatin Receptors (SSTR), or antigens (e.g., CD20, CEA) [119,120].
Representative Agents	[^68^Ga]Ga-DOTA-Folate, [^177^Lu]Lu-DOTA-Folate, [^99m^Tc]Tc-PAMA-Cobalamin [82]	[^68^Ga]Ga-PSMA-11, [^177^Lu]Lu-PSMA-617, [^177^Lu]Lu-DOTATATE, [^131^I]I-MIBG [121,122,123,124]
Key Advantages	High Biocompatibility & Low Immunogenicity: Vitamins are endogenous, naturally processed molecules. Low Cost & Accessibility: Vitamins are inexpensive and readily available. Proven Safety Profile: Generally non-toxic at physiological doses. Potential for Multimodality: Can be integrated with nanosystems for combined imaging/therapy. Broad Applicability: Targets can be overexpressed in multiple cancer types (e.g., FRα in ovarian, breast, lung).	High Target Affinity & Specificity: Often engineered for very high binding affinity to their target. Well-Established Clinical Use: Extensive clinical data and regulatory approval for several agents. Standardized Protocols: Established dosing, administration, and safety monitoring guidelines. Predictable Biodistribution: Well-characterized pharmacokinetics and clearance pathways [123]
Key Limitations & Challenges	Receptor Heterogeneity: Expression can vary between and within tumors, leading to potential false negatives. Competition with Endogenous Vitamins: High plasma levels of natural vitamins can block binding sites. Off-Target Uptake: Specific organs (e.g., kidneys for folates, liver/kidneys for B12) can show significant accumulation, posing a toxicity risk. Limited Clinical Translation: Most agents are still in preclinical or early clinical stages. Optimization of Pharmacokinetics: Requires careful engineering (e.g., linkers, albumin binders) to improve tumor-to-background ratios.	Immunogenic Potential: Peptide and antibody-based agents can provoke immune responses. Higher Production Costs: Peptide synthesis and monoclonal antibody production are expensive. Toxicities Related to Targets: Off-target binding to healthy tissues expressing the target (e.g., salivary glands for PSMA, kidneys for SSTR). Tumor Resistance Mechanisms: Tumors can downregulate target expression under therapeutic pressure [125].
Clinical Status	Primarily preclinical and early-phase clinical trials. A few agents (e.g., EC20/^99m^Tc-etarfolatide) have reached clinical studies but are not yet standard of care [126].	Clinically established and approved. Multiple agents are standard care (e.g., Lutathera^®^, Pluvicto^®^) for specific indications [127,128].
Ideal Use Case	Precision theranostics for tumors with confirmed overexpression of specific vitamin receptors, especially where conventional targets are absent or as part of combination nano therapy [129].	First-line theranostic approach for cancers with well-defined, highly expressed molecular targets like PSMA (prostate) or SSTR (neuroendocrine) [130].
Potential for Synergy	High potential for integration with nanoparticle drug delivery systems and as radiosensitizing/radioprotective adjuvants due to their biological roles.	Often used in combination with other systemic therapies (e.g., hormone therapy, chemotherapy) and increasingly with immunotherapy.

**Table 2:** Different radionuclides and targeted agents in preclinical studies with vitamins to treat various forms of cancer.

Cancer Types	Radionuclide	Targeted Agent	Results	Statistical Significance	Vitamin	Reference
Gastric cancer	^211^At	Anti-TF mAb	Dose-dependent antitumor effects with SA protection	Significant protective effect of SA (*p* < 0.05)	Sodium Ascorbate (SA)	[43]
Gastric cancer	^68^Ga	DOTA-RGD	High affinity and stability with tumor uptake	Tumor targeting significant (*p* < 0.001)	Folate	[45]
Gastric cancer	^99m^Tc	HYNIC-NHHN-FA (folate conjugate)	Low tumor uptake, primarily absorbed in stomach and intestines	Tumor targeting significant (*p* < 0.05)	Folate	[50]
Gastric cancer	^99m^Tc	HYNIC-PEG2-FA	Moderate tumor uptake; reduced uptake with excess folate	Receptor-specific targeting confirmed (*p* < 0.05)	Folate	[51]
Gastric cancer	^99m^Tc	ECG-EDA-folate	Radiolabeling efficiency: >96%; decreased uptake with excess folate	specific targeting and significant reduction in tumor uptake with excess folate (*p* < 0.05)	Folic Acid	[52]
Gastric cancer	^68^Ga	^68^Ga-NOTA-folic acid conjugate	Radiolabeling efficiency: >95%; specific uptake by KB cells; biodistribution correlated with kidney retention.	demonstrated strong correlation between tracer and kidney retention. (*p* < 0.05)	Folic Acid	[49]
Gastric cancer	^177^Lu	cm09 (albumin-binding folate conjugate)	Radiochemical efficiency, >98% Stability, >99% and Serum Protein Biding ~91%	extended blood circulation time enhanced tumor uptake. (*p* < 0.05)	Folic Acid	[44]
Gastric cancer	^18^F	α- and γ-conjugated fluorinated folate derivatives	Radiochemical purity: >95%; reduced kidney uptake by 50% with γ-regioisomers.	Binding affinity IC50: 1.4–2.2 nM; biodistribution impacted by regioisomers (*p* ≤ 0.01)	Folic Acid	[53]
Gastric cancer	^18^F	3-aza-2-[^18^F]-fluorofolic acid	High contrast imaging with minimal off-target accumulation.	demonstrated favorable properties for imaging FR-positive tumors.	Folic Acid	[54]
Gastric cancer	^18^F	[^18^F]-folic acid derivative	Surpassed previous ^18^F-labeled radiofolates; highest affinity for FR-positive tumors. radiochemical purity, >95%	Considered the most promising ^18^F-radioligand for FR-positive tumors.	Folic Acid	[55]
Gastric cancer	^18^F	[^18^F]F-fluorodeoxyglucose-folate	Enhanced hydrophilicity; demonstrated high specificity and affinity for folate receptors in cancers and inflammation.	demonstrated potential for clinical translation.	Folic Acid	[57]
Breast cancer	^177^Lu	DOTA-folate	Tumor inhibition beyond 70 days (NF9006 cells)	Significant tumor growth reduction (*p* < 0.01)	Folate	[60]
Breast cancer	^89^Zr	^89^Zirconium-labeled cobalamin (^89^Zr-Cbl)	6–10-fold higher uptake compared to excess CN-Cbl—High accumulation in kidneys and liver after 48 h.	demonstrated feasibility of tracer for tumor visualization. (*p* ≤ 0.01)	Vitamin B12, (Cobalamin)	[65]
Breast cancer	^99m^Tc	^99m^Tc(CO)3-PTX-FR-NLC,	higher uptake in folate receptor-positive organs, radiolabeling efficiency more than 90%	Tumor accumulation significant (*p* < 0.05)	Folate	[66]
Breast cancer	^177^Lu	^177^Lu-DOTA-Folate-Bombesin	High tumor uptake in breast cancer models	Significant tumor inhibition (*p* < 0.05)	Folate	[61]
Breast cancer	^99m^Tc	^99m^Tc-Bombesin-Folate	Improved breast tumor imaging and high radiochemical purity (96 ± 2.1%)	Higher tumor-to-muscle ratio (*p* < 0.05)	Folate	[62]
Breast cancer	^177^Lu	^177^Lu-Dendrimer-Folate-Bombesin-Gold Nanoparticles	Effective theranostic radiopharmaceutical and radiochemical purity (>95%)	Enhanced imaging and therapy (*p* < 0.05)	Folate	[63]
Breast cancer	^177^Lu	^177^Lu-Folate Conjugate with Iron Oxide Nanoparticles	Tumor uptake visualized via SPECT/CT	Enhanced contrast and targeting	Folate	[64]
Breast cancer	^99m^Tc	Radiolabeled Folate-Conjugated Liposomes	Increased tumor-specific imaging	High affinity for folate receptors (*p* < 0.05)	Folate	[68]
Breast cancer	^153^Sm	^153^Sm-Folate-PEI-Chitosan Nanoparticles	High radiochemical purity, (>90%)	Significant tumor localization	Folate	[69]
Breast cancer	^99m^Tc	^99m^Tc-HYNFA	Improved imaging contrast and labeling yield: 97–98%; radiochemical purity: 96–98%	Statistically significant uptake (*p* < 0.05)	Folate	[70]
Colorectal cancer	^64^Cu	B12-en-Bn-NOTA	Tumor uptake: 2.20–4.84% ID/g at 6 h	Tumor uptake significantly reduced with excess B12 (*p* < 0.05)	Vitamin B12	[74]
Colorectal cancer	^111^In	MORAb-003	Significant tumor uptake (32 ± 5% ID/g at 4 days)	Validated in small group of patients	Folate	[77]
Colorectal cancer	^89^Zr	^89^Zr-Labeled Folic Acid-Conjugated Silica	High receptor-specific binding	Strong PET signals	Folic acid	[79]
Colorectal cancer	^64^Cu	^64^Cu-Folate-Shell Cross-Linked Nanoparticles	Targeted tumor uptake	Effective biodistribution (*p* = 0.0001)	Folate	[75]
Colorectal cancer	^68^Ga	^68^Ga-HBED-CC-EDBE-Folate	High radiochemical purity (98%)	Selective tumor accumulation	Folate	[80]
Colorectal cancer	^99m^Tc	^99m^Tc-PAMA-Cobalamin	High tumor specificity in clinical study	Improved tumor imaging	Vitamin B12	[82]
Brain cancer	^18^F	[^18^F]FOL	Superior tumor-to-brain uptake ratio (TBR) compared to FDG And radiochemical purity was 97.5% ± 1.6	TBR statistically higher for [^18^F]FOL (*p* < 0.05)	Folate	[88]
Brain cancer	^18^F	[^18^F]AlF-NOTA-Folate	Good tumor uptake with reduced kidney accumulation	High specificity and rapid clearance	Folate	[89]
Brain cancer	^99m^Tc	Vitamin C-coated selenium nanoparticles (SeNPs)	Radiolabeling yield: 96 ± 2%—Stability maintained for over 6 h	demonstrated suitable target-to-non-target ratios in solid tumors.	Vitamin C	[91]
Brain cancer	^99m^Tc	Radiolabeled Folate Micellar Carriers	High efficiency for brain tumors	Enhanced SPECT imaging (*p* < 0.001)	Folate	[92]
Brain cancer	^66^Ga, ^68^Ga	^66^Ga/^68^Ga-Folate Conjugates	Tumor uptake validated via PET	High specificity	Folate	[94]
Brain cancer	^64^Cu	PET/NIR-II Fluorescence Folate Probe	Dual-modality imaging	Enhanced tumor detection (*p* < 0.0001)	Folate	[90]
Brain cancer	^99m^Tc	^99m^Tc-Radiolabeled Benzothiazole-Folate	Very high Radiochemicla purity, >99%	Significant uptake observed	Folate	[93]
Brain cancer	^68^Ga	^68^Ga-Pteroyl-Lys Conjugates	Selective folate receptor binding and favorable radiolabeling yield (>90%), higher tumor-to-non target ratio	Improved PET contrast, (*p* < 0.05)	Folate	[95]
Prostate cancer	^57^Co, ^111^In	Radiolabeled vitamin B12 derivatives	Reduced systemic distribution with improved tumor-to-blood ratio.	emphasized improvements in targeted accumulation. (*p* < 0.001)	Vitamin B12	[105]
Prostate cancer	^99m^Tc	^99m^Tc-Folate-PEG-Doxorubicin	Antitumor efficiency, radiolabeling yield greater than 90%	Enhanced tumor accumulation (*p* < 0.05)	Folate	[97]
Prostate cancer	^18^F	^18^F-Click Radiolabeled Folate	High tumor targeting And radiochemical purity, >95%.	PET-imaging validated	Folate	[102]
Prostate cancer	^99m^Tc	^99m^Tc-EC20	High radiochemical yield (>95%)	Superior tumor-to-background contrast	Folate	[99]
Prostate cancer	^99m^Tc	^99m^Tc-Labeled Vitamin C	Maximum Radiolabeling yield, 93 ± 5.0%	Favorable pharmacokinetics (*p* < 0.05)	Vitamin C	[103]
Prostate cancer	^99m^Tc	^99m^Tc-Dimeric Folic Acid	Strong FR-targeting, (IC50 = 19.06 nM)	High tumor specificity (*p* < 0.05)	Folate	[100]
Prostate cancer	^99m^Tc	^99m^Tc-Folate-Isonitrile Complexes	High radiochemical purity, >95%	Improved receptor binding And significant biodistribution (*p* < 0.05, *p* < 0.01)	Folate	[101]
Prostate cancer	^99m^Tc	^99m^Tc-Vitamin B12 Derivative	Abolished TC binding for selective uptake and radiochemical purity of PAMA-4-B12 was >95%,	Significant tumor targeting	Vitamin B12	[104]
Ovarian cancer	^125^I, ^131^I	[^125^I]-2 and [^125/131^I]-4 folate conjugates	High stability, FR-specific binding, strong tumor accumulation	Pre-injection with pemetrexed improved imaging (*p* < 0.05)	Folate	[110]
Ovarian cancer	^18^F	[^18^F]-2-folate	High radiochemical yield (>80%), purity (>97%), significant tumor uptake	Micro-PET imaging confirmed results (*p* < 0.05)	Folate	[112]
Ovarian cancer	^18^F	[^18^F]-FDG-folate and [^18^F]-8	High yield (>80%), purity (>98%), receptor specificity confirmed	Blocking experiments validated specificity (*p* < 0.05)	Folate	[113]
Ovarian cancer	^124^I	[^124^I]-SIB- and [^124^I]-SIP-folate	High radiochemical yield (>90% and >60%), purity (>98%), strong tumor targeting	Effective tumor uptake in vivo (*p* < 0.05)	Folate	[111]
Ovarian cancer	^47^Sc, ^177^Lu, ^90^Y	^47^Sc-DOTA-folate, ^177^Lu-folate, ^90^Y-folate	Tumor growth inhibition (39–43 days vs. 26 days in controls), no severe side effects	^90^Y-folate showed the highest potency (*p* < 0.05)	Folate	[114]
Ovarian cancer	^177^Lu	^177^Lu-FA-DOTA-PEG-PLGA nanoparticles	High labeling efficiency (97–98%), minimal kidney accumulation, tumor localization confirmed	Significant tumor inhibition, no major toxicity (*p* < 0.05)	Folic acid	[115]
Ovarian cancer	^188^Re	^188^Re-folate-CDDP/HAS MNP	Apoptosis rates reached 57.16% and significant inhibition tumor growth	Triple combination therapy showed strongest tumor suppression (*p* < 0.05)	Folate	[117]
Ovarian cancer	^99m^Tc	^99m^Tc-NP-PEGFA (Folate-functionalized cisplatin nanoparticles)	High cellular uptake	Enhanced blood circulation and tumor accumulation (*p* < 0.05)	Folate	[118]

## Data Availability

No new data were created or analyzed in this study.

## Supplementary Materials

The following supporting information can be downloaded at: https://www.mdpi.com/article/10.3390/jpm16010036/s1, Table S1: PRISMA checklist.

## Abbreviations

Anti-TF mAb	anti-tissue factor monoclonal antibody
^211^At	astatine-211
ADCs	antibody–drug conjugates
B9	Folate
BC	breast cancer
CIN	chromosomal instability
Cbl	Cobalamin
CDC	silica nanoparticle drug conjugate
CTLA-4	cytotoxic T-lymphocyte antigen 4
^57^Co	cobalt-57
^64^Cu	copper-64
DDS	drug delivery system
DOTA-Mal	maleimido-mono-amide-DOTA
DOX	Doxorubicin
EBV	Epstein–Barr virus
EGFR	epidermal growth factor receptor
FR	folate receptor
FRA	folate receptor alpha
FA-NPs	folate-decorated nanoparticles
^18^F	fluorine-18
FA	folic acid
FAP	fibroblast activation protein
^68^Ga	gallium-68
GC	gastric cancer
Gd	Gliadin
GRPR	gastrin-releasing peptide receptors
GBM	glioblastoma multiforme
GS	genomically stable
GEP	Gastroenteropancreatic
HYNIC	hydrazinonicotinic acid
HPLC	high-performance liquid chromatography
HGSC	high-grade serous carcinoma
^111^In	indium-111
^124^I	iodine-124
^125^I	iodine-125
^131^I	iodine-131
LET	linear Energy Transfer
^177^Lu	lutetium-177
mAb	monoclonal antibody
MT1-MMP	membrane type-1 matrix metalloproteinase
MRI	magnetic resonance imaging
MDR	multidrug resistance
MSI	microsatellite instability
mCRPC	metastatic castration-resistant prostate cancer
NLCs	nanostructure lipid carriers
NET	neuroendocrine neoplasms
OC	ovarian cancer
PET	positron emission tomography
PEG	polyethylene glycol
PSMA	prostate-specific membrane antigen
PAMAM	poly(amidoamine)
PTX	Paclitaxel
PRRT	peptide receptor radionuclide therapy
^188^Re	rhenium-188
SA	sodium ascorbate
^47^Sc	scandium 47
^153^Sm	samarium-153
SeNPs	selenium nanoparticles
SNPs	silica nanoparticles
SCKs	shell cross-linked nanoparticles
SMWs	micro-scale silica wires
SPECT	Single-photon emission computed tomography
SSTR	somatostatin receptor
TRT	targeted radionuclide therapy
^99m^Tc	technetium-99m
TPP	triphenylphosphonium
TLC	Thin-layer chromatography
TC	transcobalamin
TC-II	transcobalamin II
TBR	tumor-to-brain uptake ratio
UPN	uncoated nanoparticles
VEGFR	Vascular endothelial growth factor
WHO	world health organization
^90^Y	yttrium-90
^89^Zr	zirconium-89
